# Direct and indirect resource use, healthcare costs and work force absence in patients with non‐infectious intermediate, posterior or panuveitis

**DOI:** 10.1111/aos.12987

**Published:** 2016-03-02

**Authors:** Jennifer E. Thorne, Martha Skup, Namita Tundia, Dendy Macaulay, Cindy Revol, Jingdong Chao, Avani Joshi, Andrew D. Dick

**Affiliations:** ^1^ Department of OphthalmologyJohns Hopkins School of MedicineBaltimoreMDUSA; ^2^ Department of EpidemiologyCenter for Clinical TrialsJohns Hopkins Bloomberg School of Public HealthBaltimoreMDUSA; ^3^ AbbVie Inc.North ChicagoILUSA; ^4^ Analysis Group, Inc.New YorkNYUSA; ^5^ Clinical SciencesUniversity of BristolBristolUK; ^6^ National Institute for Health Research Biomedical Research CentreMoorfields Eye Hospital and Institute of OphthalmologyLondonUK

**Keywords:** direct costs, indirect costs, non‐infectious, persistent, uveitis, work disability

## Abstract

**Purpose:**

To ascertain resource use, costs and risk of workforce absence in non‐infectious uveitis cases versus matched controls.

**Methods:**

In a retrospective claims analysis of employees in the United States, prevalent (*N *= 705) and incident (*N *= 776) cases 18–64 years old with ≥2 diagnoses of non‐infectious intermediate, posterior or panuveitis were matched 1:1 to controls without uveitis. Persistent prevalent cases (treated for ≥90 days, *N *= 112) also were analysed. Outcomes were annual direct resource use and costs associated with inpatient stays; emergency department, outpatient and ophthalmologist/optometrist visits; and prescription drugs. Indirect resource use and costs associated with work loss from disability and medically related absenteeism also were compared. Multivariate regression assessed cost differences between cases and controls.

**Results:**

Cases had significantly (p < 0.05) more medical resource use versus controls including 0.4 versus 0.2 emergency visits and 16.5 versus 7.6 outpatient/other visits. Cases used more prescription drugs (7.8 versus 4.1) and had more disability days (10.3 versus 4.6), medically related absenteeism days (8.5 versus 3.8), and work loss days (18.7 versus 8.4) than controls (all p < 0.05). Total direct ($12 940 versus $3730) and indirect ($3144 versus $1378) costs were higher in cases than controls (all p < 0.05). Results for persistent cases suggested greater utilization and associated cost and work loss burden. Compared with controls, cases had significantly greater risks of workforce absence, leave of absence and long‐term disability (all p < 0.05).

**Conclusion:**

Non‐infectious intermediate, posterior or panuveitis, particularly persistent disease, is associated with substantial medical and work loss costs suggesting an unmet need for more effective treatments.

## Introduction

Uveitis is a broad term for inflammation inside the eye. Symptoms of uveitis vary by anatomic location and include redness, pain, photosensitivity and blurred/reduced vision. Uveitis can be infectious or non‐infectious in origin, with non‐infectious disease predominating in the developed world (Miserocchi et al. 2013). Non‐infectious uveitis may be idiopathic or associated with systemic autoimmune diseases (Rothova et al. 1996; Bodaghi et al. 2001; Chang & Wakefield 2002; Nguyen et al. 2011; Barisani‐Asenbauer et al. 2012; Chu et al. 2013; Levin et al. 2014). Regardless of the aetiology, persistent intra‐ocular inflammation can lead to structural ocular complications and visual disability (Rothova et al. 1996; Durrani et al. 2004a,b; Nguyen et al. 2011; Barisani‐Asenbauer et al. 2012; Chu et al. 2013; Pan et al. 2014). The Standardization of Uveitis Nomenclature (SUN) guidelines classify uveitis by anatomic location as anterior, intermediate, posterior or panuveitis (Jabs et al. 2005); hereafter, non‐infectious intermediate, posterior and panuveitis will be referred to collectively as NIIPPU. Compared with anterior disease, which is the most common non‐infectious form (Chang & Wakefield 2002; Gritz & Wong 2004; Acharya et al. 2013; Miserocchi et al. 2013), NIIPPU is more often associated with a poorer prognosis (Chang & Wakefield 2002; Barisani‐Asenbauer et al. 2012).

Overall, uveitis is estimated to cause approximately 5–15% of blindness in the United States and Europe, making it approximately the 5th or 6th leading cause of preventable blindness (Nussen‐blatt^1990^; Rothova et al. 1996; Suttorp‐Schulten & Rothova 1996; Durrani et al. 2004a; Miserocchi et al. 2013). Vision loss due to NIIPPU can negatively affect patients' mental and physical health (Schiffman et al. 2001; Murphy et al. 2005, 2007; Miserocchi et al. 2010; Qian et al. 2012). Approved treatments for NIIPPU include local and systemic corticosteroid therapy and regional therapies such as surgically placed corticosteroid implants, although conventional steroid‐sparing immunosuppressive agents and biologics also are used in clinical practice (Multicenter Uveitis Steroid Treatment Trial Research Group et al. 2011; Gallego‐Pinazo et al. 2013).

Disability associated with visual impairment also can adversely affect patients' ability to work and may lead to absenteeism or early exit from the workforce. Because NIIPPU has relatively early onset and the incidence is greatest in working age people (Suttorp‐Schulten & Rothova 1996; Durrani et al. 2004a; Acharya et al. 2013), the costs to society in terms of medical resource use and work disability could be greater than those observed among patients with blindness or visual impairment associated with age‐related eye diseases. Nevertheless, current data on the economic burden of non‐infectious uveitis are limited, aside from a study by Chu et al. (2013) that reported direct costs in a population that included patients with anterior disease. To our knowledge, the potential economic and resource burden specifically for an NIIPPU population has not been reported to date. To augment the available literature, we assessed direct (medical service and prescription drug) and indirect (work loss) resource use and costs for privately insured United States (US) employees with NIIPPU and compared them to matched controls without uveitis; an analysis of persistent NIIPPU cases also was conducted. In addition, risks of workforce absence associated with NIIPPU were assessed.

## Materials and Methods

### Patient sample and data source

**Figure 1:**
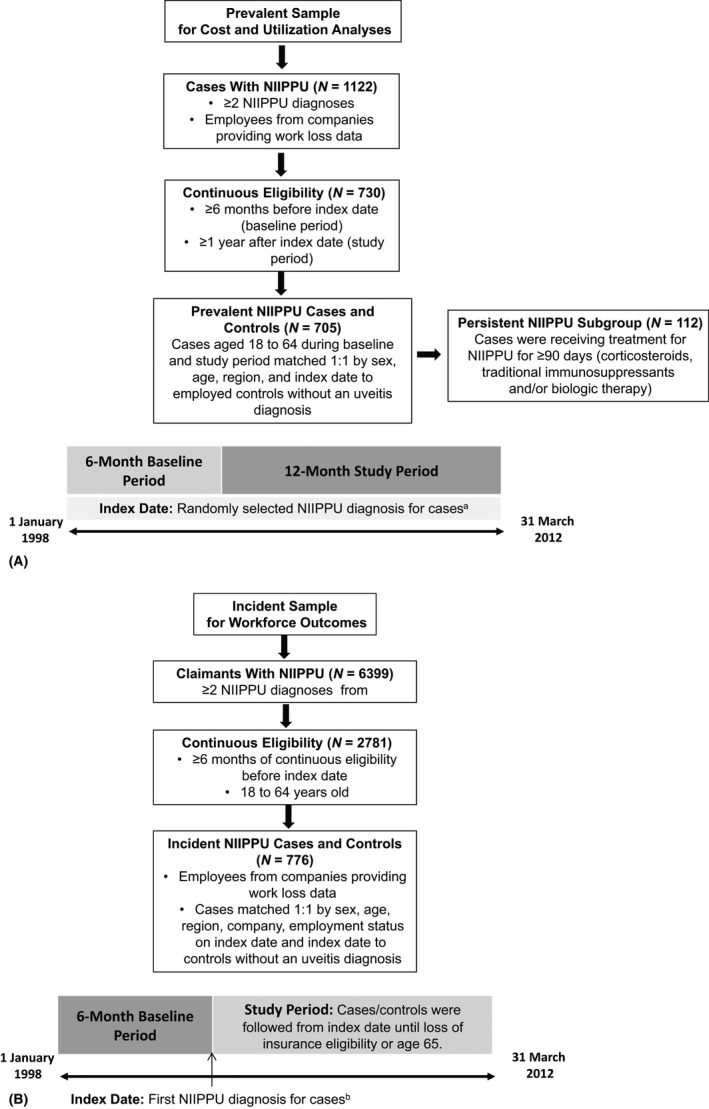
Sample selection. (A) Prevalent sample for costs and utilization analyses. ^a^Includes NIIPPU cases at a variety of points in their disease (e.g. recently diagnosed patients and patients who have NIIPPU for a longer period of time); it is possible that NIIPPU patients in the prevalent sample had an NIIPPU diagnosis during the baseline period. Controls were assigned the index date of their matched NIIPPU case. (B) Incident Sample for Workforce Outcomes. ^b^Controls were assigned the index date of their matched NIIPPU case. NIIPPU, non‐infectious intermediate, posterior or panuveitis.

Two different samples were extracted from the OptumHealth Reporting and Insights database from 1 January 1998 through 31 March 2012 (OptumHealth website 2015). A cross‐sectional sample of prevalent NIIPPU cases (Fig. 1A) was selected for analyses of utilization and associated costs, and a longitudinal sample of incident NIIPPU cases (Fig. 1B) was selected for analyses of workforce outcomes. Cases 18 to 64 years of age were identified using International Classification of Diseases, Ninth Revision, Clinical Modification (ICD‐9‐CM) codes for non‐infectious intermediate, posterior or panuveitis [360.12 (panuveitis), 362.12 (exudative retinopathy), 362.18 (retinal vasculitis), 363.0x (focal chorioretinitis and focal retinochoroiditis), 363.10‐13 and 363.15 (disseminated chorioretinitis and disseminated retinochoroiditis), 363.2x (other and unspecified forms of chorioretinitis and retinochoroiditis, including pars planitis) and 364.24 (Vogt‐Koyanagi syndrome)]. Both primary and secondary NIIPPU diagnoses were included, and ≥2 diagnoses were required for confirmation of the condition. The selected codes were modified from Reeves et al. (2006) to exclude anterior and infectious uveitic diagnoses. In the prevalent sample, a subgroup of persistent NIIPPU cases was defined as those receiving treatment for NIIPPU for ≥90 days (corticosteroids, traditional immunosuppressants and/or biologic therapy) (Jabs et al. 2005).

The OptumHealth database includes 16.4 million privately insured individuals from a diversity of industry sectors, such as financial services, manufacturing, telecommunications, energy and the food/beverage industry. Medical and drug claims and eligibility data are available for the primary policyholders and all beneficiaries. Additionally, short‐ and long‐term disability claims for employees are available for a subset of the companies. The database is compliant with the Health Insurance Portability and Accountability Act. Ethics approval was not required for this study because the data analysed were de‐identified records from an administrative insurance database.

### Outcome measures

Available data included employees' benefit eligibility, medical and pharmacy service claims, disability claims, plan‐specific length of waiting period before short‐term disability and salary information. Direct resource outcomes and associated costs included hospitalizations, emergency department (ED) visits, outpatient/other visits, ophthalmologist/optometrist visits and prescription drug use. Indirect resource outcomes included disability days associated with extended absence from work due to short‐ or long‐term disability and medically related absenteeism days associated with medical claims occurring during days of work. Outcomes associated with leaving the workforce were time to leave of absence, short‐term disability, long‐term disability and leaving for any of these reasons plus early retirement.

### Data analysis

NIIPPU cases were matched 1:1 on sex, age, region and index date to controls without uveitis. Baseline demographic characteristics, ocular and autoimmune comorbidities, and Charlson Comorbidity Index (CCI) were compared descriptively between cases and controls using McNemar's tests for categorical variables and Wilcoxon signed‐rank tests for continuous variables. Prevalent NIIPPU cases and their matched controls who were eligible for the utilization and cost analyses were required to have continuous eligibility for ≥6 months before and ≥1 year after the index date (Fig. 1A). The index date for cases was a randomly selected NIIPPU diagnosis any time during the baseline or follow‐up period. This approach allowed selection of a sample of NIIPPU patients who were at a variety of points in their disease course. Direct medical utilization and costs were disaggregated by place of service and included the following categories: inpatient, ED, and outpatient/other; prescription drug utilization and costs included all claims for prescription drugs during the study period. Direct healthcare costs were calculated based on all payments to providers for medical services and prescription drugs. Indirect resource use and costs included both work loss owing to disability and medically related absenteeism. Days of disability were computed by identifying the total time covered by short‐ and long‐term disability claims. Medically related absenteeism days were imputed based on use of medical services during business days (e.g. an office visit or a hospital inpatient visit during Monday through Friday), as well as the waiting period in advance of the start of short‐term disability (e.g., five missed days of work due to illness). The methodology assumed that each hospitalization day and ED visit accounted for a full day of work loss, whereas each outpatient/other visit accounted for half a day of work loss. Disability costs were based on actual employer disability payments, and medically related absenteeism costs were calculated based on individual employee wage information and days of medically related absenteeism. All costs were adjusted to 2012 US dollars. Direct and indirect resource use and costs incurred during the study period were compared between the two cohorts using Wilcoxon signed‐rank or McNemar's tests. Multivariate regression assessed key cost differences between cases and controls, with adjustment for age, sex, region, index year (full sample only) and CCI.

Cases eligible for the risk of leaving the workforce analyses were required to be active employees in a company providing work loss data on the date of first NIIPPU diagnosis; cases and controls were followed from index date until loss of insurance eligibility or age 65 (Fig. 1B); the index date for cases was the first NIIPPU diagnosis and controls were assigned the index date of their matched case. Time‐to‐event Kaplan–Meier analysis and log‐rank tests were used to compare risks of leaving the workforce between cases and controls. The time to each event was calculated as the time from the index date to the earliest event of that type; cases and controls who did not have the event after the index date were censored at the last day of follow‐up (i.e. the end of eligibility or when the participant turned 65 year of age). Cox proportional hazards regressions with adjustment for differences in age, sex, region, CCI score, presence of autoimmune diseases and presence of human immunodeficiency virus/acquired immunodeficiency syndrome (HIV/AIDS) were used to estimate hazard ratios for NIIPPU cases relative to controls during the entire duration of follow‐up, at 1 year and at 5 years.

## Results

### Baseline data

For the prevalent NIIPPU analysis (i.e. resource use and cost analysis), the mean age of the NIIPPU cases (*N *= 705) and matched controls without uveitis (*N *= 705) was 45.3 years, and 63% were men. For all of the selected baseline ocular and most of the autoimmune comorbidities, significantly greater frequencies were observed for the NIIPPU cohort compared with matched controls (Table S1). Retinal disorders (12% for cases, 0.1% for controls), visual disturbances (11% for cases, 0.7% for controls), glaucoma (7% for cases, 2% for controls), and cataract (6% for cases, 0.4% for controls) were the most common ocular comorbidities among cases (all p < 0.0001, McNemar's test). Sarcoidosis (3% for cases, 0.1% for controls; p < 0.0001) and rheumatoid arthritis (2% for cases, 0.4% for controls; p < 0.0017) were the most common baseline autoimmune comorbidities. All ocular and autoimmune comorbidities, except for glaucoma, were present in ≤1% of the matched control population. Mean CCI scores were significantly greater in patients with NIIPPU versus controls (0.9 versus 0.1; p < 0.0001, Wilcoxon signed‐rank test).

Mean age in the prevalent, persistent NIIPPU subgroup (*N *= 112) was 47.8 years and 58% were men. Baseline ocular complications, except for blindness, were significantly more common in cases versus controls (all p ≤ 0.01; Table S2). Retinal disorders (21% versus 0%) were the most common, followed by glaucoma (15% versus 1%), visual disturbances (13% versus 2%) and cataract (12% versus 0%). Similar to the full NIIPPU prevalent sample, most the selected autoimmune comorbidities were significantly more common among cases with persistent NIIPPU versus controls. Mean CCI was significantly higher for persistent cases (0.75) versus controls (0.21) (p < 0.05).

For the incident NIIPPU analysis (i.e., work loss analysis), mean age of the NIIPPU cases (*N *= 776) and matched sample (*N *= 776) was 44.7 years and 62% were men. All ocular comorbidities were statistically significantly more common in cases compared with the matched controls, with retinal disorders (9% for cases, 0% for controls), visual disturbances (10% for cases, 0.5% for controls) and glaucoma (7% for cases, 1% for controls) being the most common (all p < 0.0001; Table S1). Across autoimmune comorbidities at baseline, incident NIIPPU cases had significantly (all p < 0.05) higher frequencies of any selected autoimmune comorbidity overall (10% cases, 2% controls), systemic vasculitis (2% cases, 0% controls), sarcoidosis (2% cases, 0% controls), spondyloarthritis (1% cases, 0.1% controls) and multiple sclerosis (1% cases, 0.1% controls) versus their matched controls. Mean CCI scores for incident NIIPPU patients were significantly greater than their matched controls (0.8 versus 0.2; p < 0.0001).

### Healthcare resource utilization

**Figure 2:**
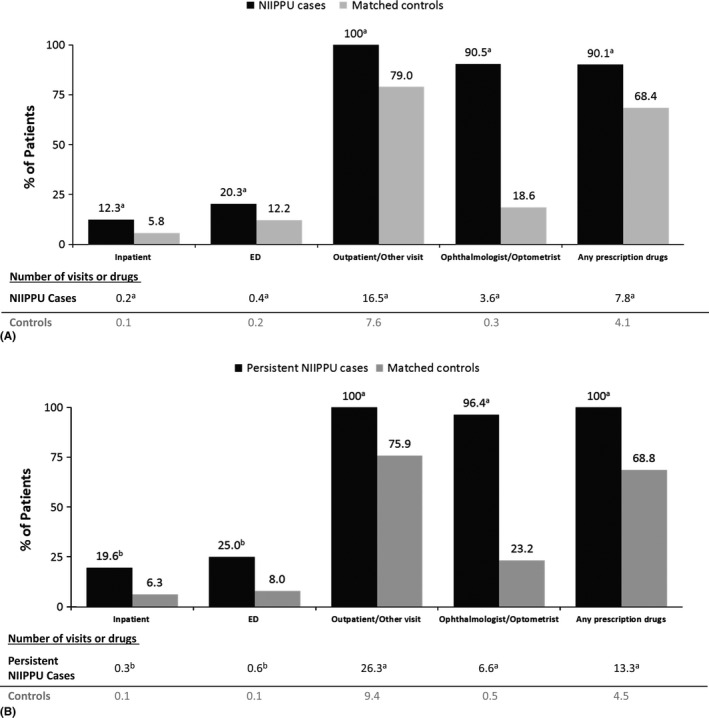
Direct Healthcare Resource Use. (A) Full NIIPPU sample. (B) Persistent NIIPPU subgroup. ^a^ *P* < 0.0001. ^b^ *P* < 0.05. NIIPPU, non‐infectious intermediate, posterior or panuveitis.

Annual direct healthcare resource utilization was greater for patients with NIIPPU than for their matched controls without uveitis, both in terms of the percentage of patients requiring direct healthcare and/or prescription drugs and the mean number of visits and/or prescriptions (Fig. 2A). Specifically, cases had higher incidences of inpatient (12% versus 6%), ED (20% versus 12%), and outpatient/other (100% versus 79%) visits compared with controls (all p < 0.0001, Wilcoxon signed‐rank test). In addition, 91% of cases had claims for prescription drugs compared with 68% of controls (p < 0.0001). Cases had 16.5 outpatient visits on average compared with 7.6 visits for controls, and cases were treated with an average of 7.8 prescription drugs compared with 4.1 prescription drugs for controls (both p < 0.0001); length of hospital stay was not significantly different between cases (6.7 days) and controls (4.2 days). The pattern of results was similar for the persistent NIIPPU subgroup (Fig. 2B). Although statistical comparisons between the full NIIPPU sample and the persistent NIIPPU subgroup were not performed, greater percentages of NIIPPU cases in the persistent population required direct healthcare and/or prescription drugs and more visits and prescriptions.

**Figure 3:**
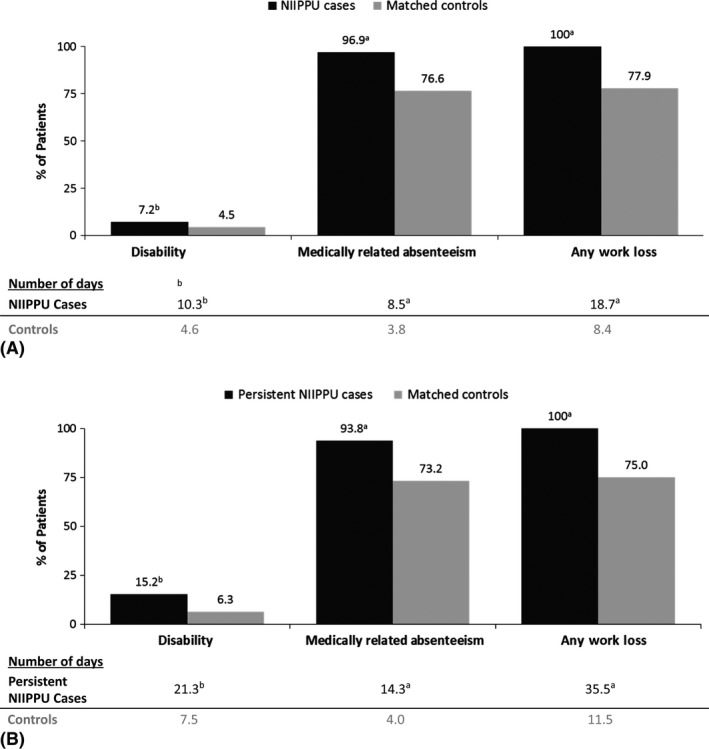
Indirect Healthcare Assessment: Work disability. (A) Full NIIPPU sample. (B) Persistent NIIPPU subgroup. ^a^ *P* < 0.0001. ^b^ *P* < 0.05. NIIPPU, non‐infectious intermediate, posterior or panuveitis.

With respect to indirect healthcare resource utilization in the full NIIPPU sample (Fig. 3A), patients with NIIPPU on average had a greater number of disability days (10.3 versus 4.6; p < 0.05), medically related absenteeism days (8.5 versus 3.8; p < 0.0001), and total days of work loss (18.7 versus 8.4; p < 0.0001) compared with their matched controls without uveitis. In the persistent NIIPPU subgroup, 15% of cases versus 6% of controls had work disability (21.3 days versus 7.5 days; p < 0.05); a total of 35.5 days of work were lost for persistent NIIPPU cases versus 11.5 for the matched controls (p < 0.0001) (Fig. 3B).

### Healthcare costs

**Figure 4:**
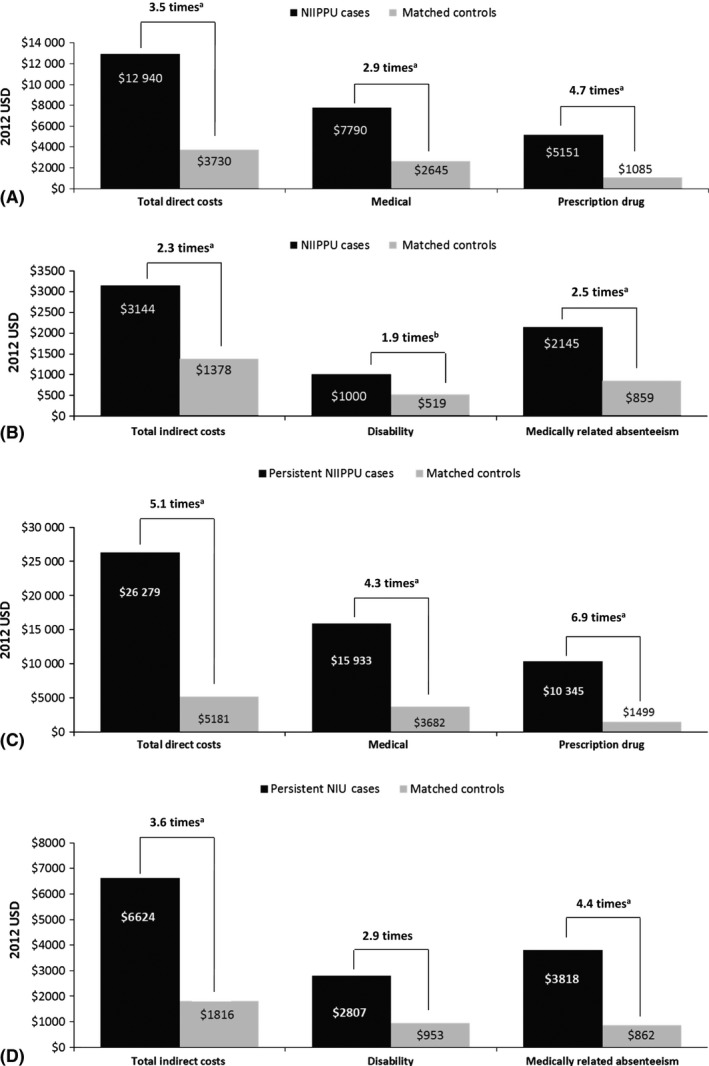
Costs for patients with NIIPPU versus controls. (A) Unadjusted direct costs: Full NIIPPU sample. (B) Unadjusted indirect costs: full NIIPPU sample. (C) Unadjusted direct costs: persistent NIIPPU subgroup. (D) Unadjusted indirect costs: persistent NIIPPU subgroup.^a^ *P* < 0.0001. ^b^ *P* < 0.05. NIIPPU, non‐infectious intermediate, posterior or panuveitis; USD, US dollars.

In the unadjusted cost analyses, total direct costs were 3.5 times higher for NIIPPU cases than for controls (p < 0.0001; Fig. 4A); medical costs ($7790 versus $2645) were greater than pharmacy costs ($5151 versus $1085). Outpatient costs accounted for $5975 of the medical costs for NIIPPU cases and $1997 for controls. Total indirect costs were 2.3 times higher (p < 0.0001, multivariate regression; Fig. 4B), and costs associated with medically related absenteeism costs were greater than disability costs (Fig. 4B). After adjustment for potentially confounding baseline characteristics, total direct ($11 424 versus $5090) and indirect ($3034 versus $1510) costs were significantly higher for NIIPPU cases than for controls (both p < 0.0001; Figure S1).

For the persistent NIIPPU subgroup, annual unadjusted mean direct healthcare costs were 5.1 times greater for cases versus controls ($26 279 versus $5181; p < 0.0001) (Fig. 4C). These costs included medical costs (inpatient [$3628 versus $586], outpatient [$12 038 versus $3065], ophthalmology/optometry [$3486 versus $830, emergency [$267 versus $31]) and prescription drug costs ($10 345 versus $1499), all of which were significantly greater for cases versus controls (all p < 0.05). Total indirect costs were 3.6 times greater for persistent NIIPPU cases versus controls ($6624 versus $1816; p < 0.0001) (Fig. 4D), with medically related absenteeism costs being 4.4 times greater for persistent NIIPPU cases compared with their matched controls. Adjusted total direct costs were $35 739 for persistent NIIPPU cases and $7670 for the matched controls (p < 0.0001); adjusted total indirect costs were $6902 and $1612, respectively (p < 0.0001; Figure S2).

### Risk of workforce absence and disability

**Figure 5:**
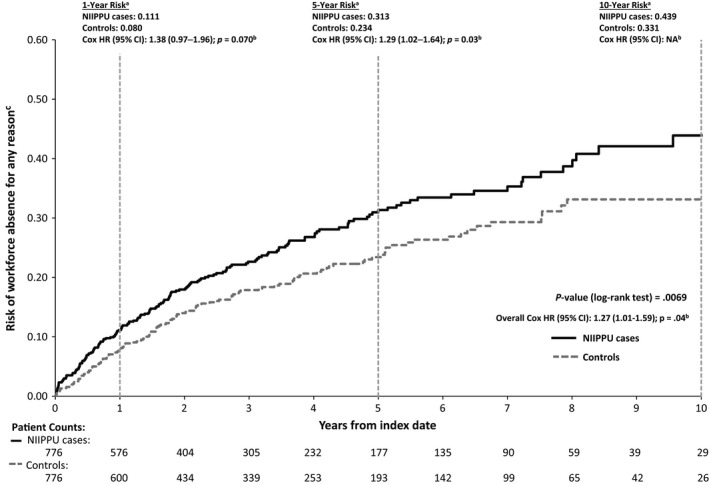
Survival Analyses for Risk of Leaving the Workforce, All‐Cause. ^a^Kaplan–Meier analyses encompassed the entire follow‐up period; however, only the first 10 years are displayed. ^b^HRs and 95% CIs were estimated from adjusted Cox regression analyses, controlling for age, sex, region, CCI, autoimmune disease and HIV/AIDS; ratios represent the hazard for NIIPPU cases relative to controls. ^c^All‐cause was defined as events of leave of absence, short‐term disability, long‐term disability or early retirement. AIDS, acquired immunodeficiency syndrome; CCI, Charlson Comorbidity Index; CI, confidence interval; HIV, human immunodeficiency virus; HR, hazard ratio; NIIPPU, non‐infectious intermediate, posterior or panuveitis.

In unadjusted Kaplan–Meier analyses, incident NIIPPU cases were at significantly greater risk of leaving the workforce for any reason over the course of follow‐up compared with their matched controls (p* *= 0.007, log‐rank test; Fig. 5). The 1‐, 5‐ and 10‐year probabilities of leaving the workforce in NIIPPU cases were 11%, 31% and 44%, respectively, compared with 8%, 23%, and 33% for controls. Compared with controls, cases also had significantly greater risks of leave of absence (p* *= 0.03; Figure S3) and long‐term disability (p* *= 0.01; Figure S4). Risk of short‐term disability was not statistically significant (Figure S5). In Cox regression models controlling for patient demographics and clinical characteristics, cases were significantly more likely than controls to leave the workforce for any reason during the entire course of follow‐up (hazard ratio* *= 1.27; p* *= 0.04); the 5‐year risk was also statistically significant with a hazard ratio of 1.29 (p* *= 0.03).

## Discussion

The present study highlights the potentially under‐recognized health impact of NIIPPU from the perspective of direct medical resource use, work loss outcomes and associated costs. With an estimated prevalence of 58–115 cases per 100 000 persons in the United States (Gritz & Wong 2004; Suhler et al. 2008; Acharya et al. 2013), NIIPPU is not particularly common; however, sight‐threatening complications, including macular oedema, glaucoma, visual disturbances and cataracts, are common in these patients (Rothova et al. 1996; Durrani et al. 2004a,b; Levin et al. 2014; Tomkins‐Netzer et al. 2014; Jones 2015). The majority of patients (63%) in this study were men. In contrast, several epidemiological studies of uveitis (all types) have reported that the majority of patients (52–57%) were women (Gritz & Wong 2004; Acharya et al. 2013; Bajwa et al. 2015). The reason for the difference in demographics in our study is directly related to selection of the study sample. In order to evaluate the risk of leaving the workforce among patients with NIIPPU, the study sample was restricted to active employees (i.e. primary policyholders) in companies providing work loss data in the claims database. From overall labour force demographics in the United States, it is expected that selecting a population with available workforce data would result in a sample of patients that is predominantly male. Indeed, in an incident sample of uveitis cases identified before restricting the sample to active employees, the sample was 45% male. Restricting the sample to active employees with work loss data resulted in a smaller sample size that was 63% male. It should be noted that the matched controls in the work loss and indirect cost analyses were also required to be active employees in companies providing work loss data. In this study, patients with NIIPPU had greater baseline prevalences of ocular complications and systemic comorbidities, including autoimmune diseases and conditions such as diabetes with complications, cerebrovascular disease, rheumatic diseases and HIV/AIDS than matched controls. Likewise, medical costs and prescription drug costs during the 1‐year study period were 2.9 and 4.7 times higher, respectively, for the NIIPPU cohort. With medical costs 4.3 times higher and prescription drug costs 6.9 times higher for the persistent NIIPPU subgroup versus matched controls, these findings suggest even greater burden for persistent disease.

Quantification of the indirect cost burden associated with workforce absence in patients with NIIPPU is a unique aspect of the present study. NIIPPU often affects patients during their productive working years (Durrani et al. 2004a; Acharya et al. 2013); thus, disease‐related work absenteeism and disability is an important measure of the potential economic consequences of the disease. Greater durations of medically related work absenteeism among NIIPPU cases in translated to associated indirect costs that were 2.5 times greater than those for controls (4.4 times greater for persistent NIIPPU), and patients with NIIPPU also were more likely to be on short‐ or long‐term disability. These findings underscore the importance of pursuing optimal treatment initiatives to manage the symptoms and comorbidities of this sight‐threatening disease, thereby potentially increasing workforce participation and reducing the socio‐economic burden of NIIPPU.

To our knowledge, the present study is the first to examine utilization, costs and work loss outcomes specifically in a population with NIIPPU. Chu et al. (2013) recently reported costs and utilization associated with non‐infectious uveitis in a privately insured population in the United States. However, the Chu et al. (2013) report differed from the present analysis in that it included patients with anterior uveitis; patients were required to be receiving treatment with corticosteroids, immunosuppressants or biologics without a control group who did not have uveitis; and work outcomes and indirect costs were not reported. Although comparison of results should be interpreted with caution for these reasons, Chu et al. (2013) reported annual direct costs (in 2009 US dollars), including study drug costs, of $13 728 for patients treated with corticosteroids, $21 108 for patients treated with immunosuppressants and $32 268 for patients treated with biologics. In comparison, annual utilization costs for diseases such as diabetes and hypertension have been estimated to be $12 192 and $8676, respectively (2009 US dollars); thus, the direct economic burden of NIIPPU ($12 940 unadjusted, $11 424 adjusted) is at least as great as that of these more common conditions (Laliberté et al. 2009; Chu et al. 2013). Moreover, total direct costs observed in the present study for persistent NIIPPU ($26 279 unadjusted, $35 739 adjusted) exceed those reported for other autoimmune diseases such as Crohn's disease ($18 022–$18 932), moderate‐to‐severe ulcerative colitis ($23 085, adjusted) and rheumatoid arthritis ($13 012, adjusted) (Yu et al. 2008; Kawatkar et al. 2012; Cohen et al. 2015).

The present study was subject to the known limitations of retrospective studies based on healthcare claims data, including possible database errors or omission of relevant claims. Using claims data to identify patients with NIIPPU may overestimate the sample size. For example, one of the ICD‐9 codes (362.12 [exudative retinopathy]) used to identify NIIPPU cases in this study may include non‐inflammatory lesions with a range of structural vascular pathology, such as Coat's disease. In addition, clinical data were limited, and the diagnoses of NIIPPU were not confirmed by ophthalmologic examinations. Although we controlled for several clinical and demographic factors in the analyses, claims data may not capture other potentially relevant factors. Finally, uveitis is frequently associated with various immunological systemic diseases (Barisani‐Asenbauer et al. 2012; Pan et al. 2014). It is possible that some of the workforce loss seen in this study is related to underlying systemic conditions. On the other hand, it is also possible that uveitis itself increases the likelihood of work loss in patients with idiopathic disease. Because systemic comorbidities are an integrated part of the burden of uveitis, this study focused on results associated with the overall burden of uveitis without separating idiopathic from systemic autoimmune disease‐related uveitis. Future research may be useful to further assess the differential burden between these two types of uveitis.

Strengths of claims data analyses include that they allow for identification of relatively large samples of cases that can be matched to controls that represent a cross‐sectional sample with health conditions and comorbidities other than the disease under study. In addition, claims data provide good representation of the insured working US population; however, results may not be generalizable to uninsured and elderly populations. Claims database also include patients treated at both primary care and specialty centres, which reduces referral bias.

In conclusion, NIIPPU was associated with substantial direct healthcare costs, both medical and pharmacy, compared with matched controls without uveitis. NIIPPU cases also had greater indirect costs associated with increased work disability and absenteeism. Persistent NIIPPU was associated with greater baseline ocular and autoimmune comorbidities, resource use and cost burdens. Together, these findings underscore the unmet need for additional therapies, for currently corticosteroids are the only approved treatment.

## Supporting information


**Figure S1.** Adjusted total direct and indirect costs: full NIIPPU sample.
**Figure S2.** Adjusted total direct and indirect costs: persistent niippu subgroup.
**Figure S3.** Risk of workforce absence: leave of absence.
**Figure S4.** Risk of workforce absence: long‐term disability.
**Figure S5.** Risk of workforce absence: short‐term disability.

**Table S1.** Comparison of comorbidity profiles: full niippu samples^a^.
**Table S2.** Comparison of comorbidity profiles: prevalent sample, persistent cases^a^.
