# Supplementary material for: Direct and indirect resource use, healthcare costs and work force absence in patients with non‐infectious intermediate, posterior or panuveitis
Source: Acta Ophthalmol. 2016 Mar 2;94(5):e331–9. doi: 10.1111/aos.12987 (PMC5069656; doi:10.1111/aos.12987)
Supplement: Supplementary file 1 — Figure S1. Adjusted total direct and indirect costs: full NIIPPU sample. Figure S2. Adjusted total direct and indirect costs: persistent niippu subgroup. Figure S3. Risk of workforce absence: leave of absence. Figure S4. Risk of workforce absence: long‐term disability. Figure S5. Risk of workforce absence: short‐term disability. [file AOS-94-e331-s001.pdf]

eFigure 1. Adjusted Total Direct and Indirect Costs: Full NIIPPU Sample

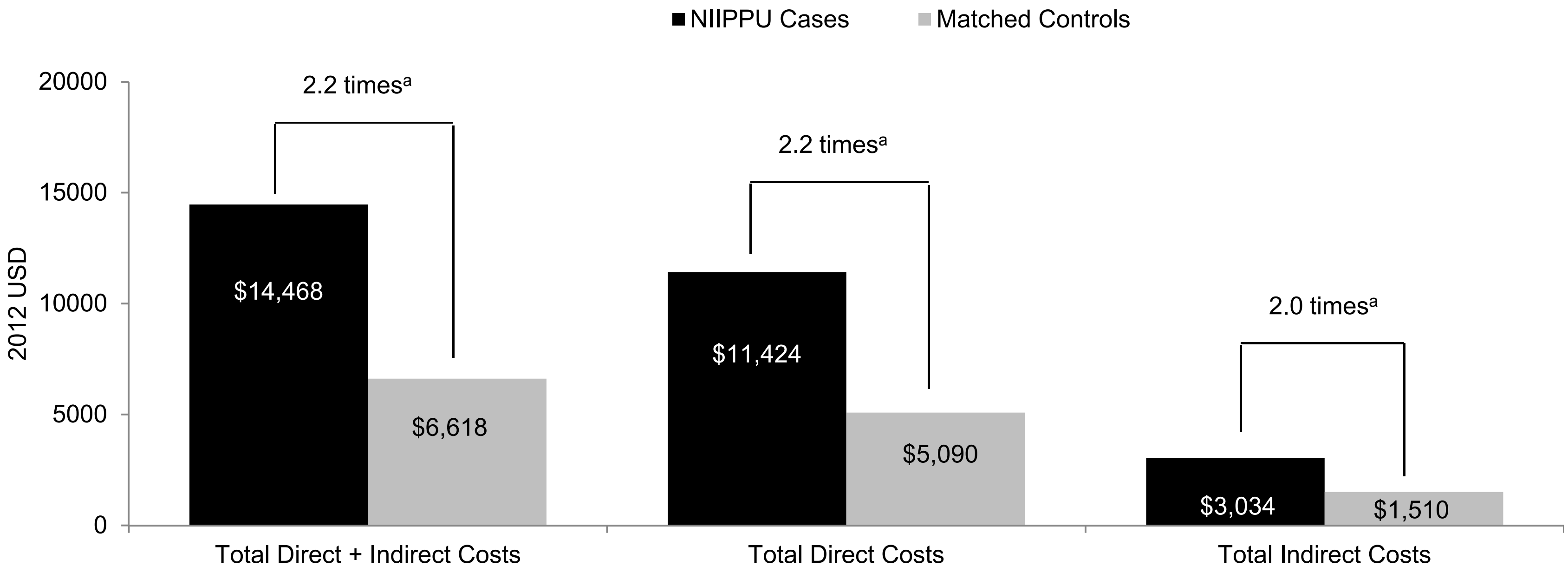

[a]$P < .0001$.
NIIPPU, non-infectious intermediate, posterior, or panuveitis; USD, US dollars.

eFigure 2. Adjusted Total Direct and Indirect Costs: Persistent NIIPPU Subgroup

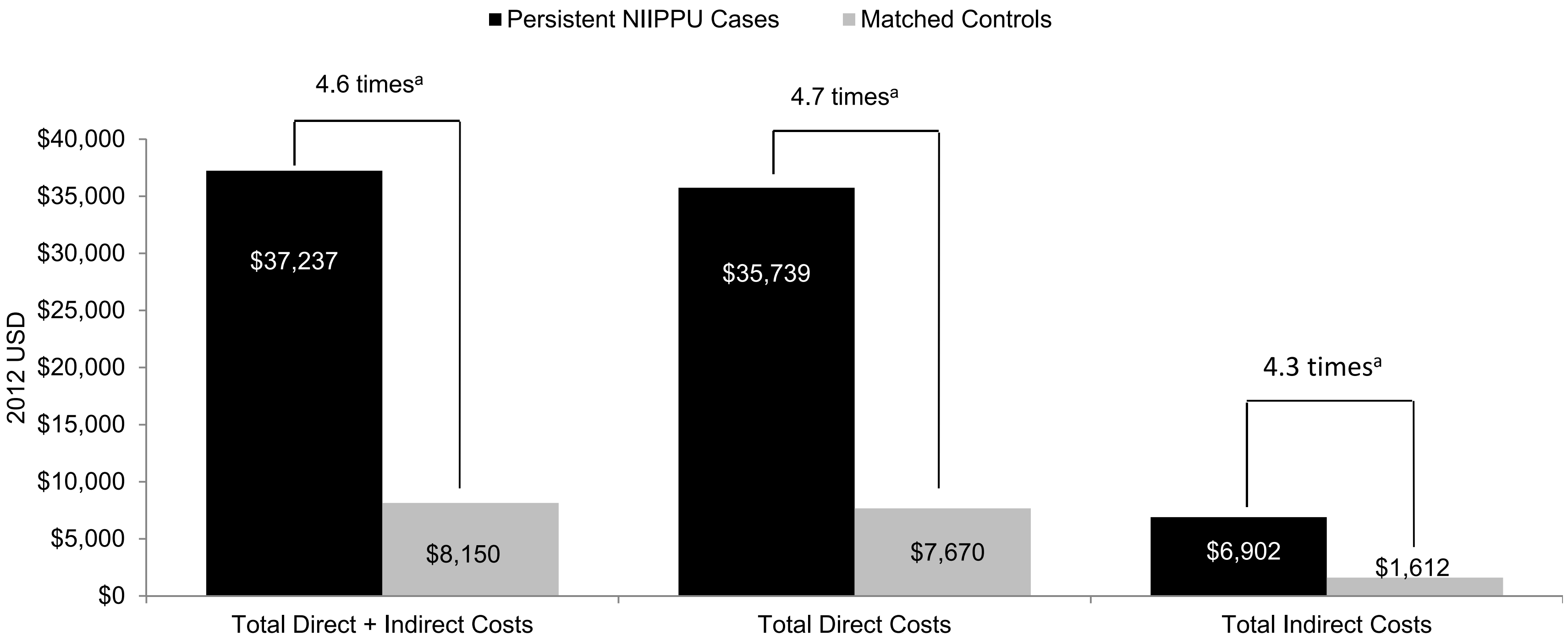

[a]$P < .0001$.

NIIPPU, non-infectious intermediate, posterior, or panuveitis; USD, US dollars.

eFigure 3. Risk of Workforce Absence: Leave of Absence

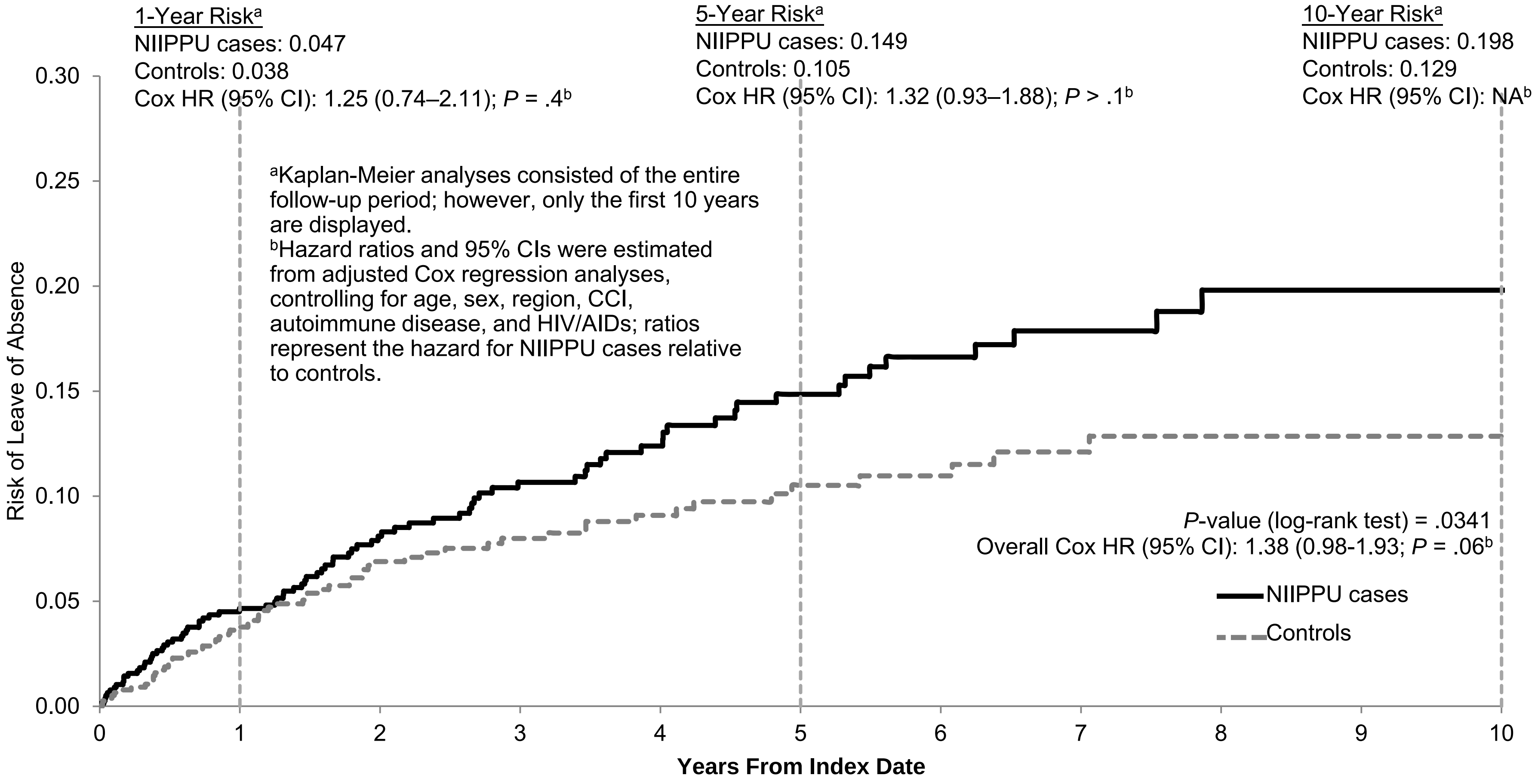

Patient counts:

NIIPPU cases:	776	615	448	346	271	209	161	108	71	50	36
Controls:	776	627	468	379	289	222	170	122	81	50	30

AIDS, acquired immunodeficiency syndrome; CCI, Charlson Comorbidity Index; CI, confidence interval; HIV, human immunodeficiency virus; HR, hazard ratio; NIIPPU, non-infectious intermediate, posterior, or panuveitis.

eFigure 4. Risk of Workforce Absence: Long-Term Disability

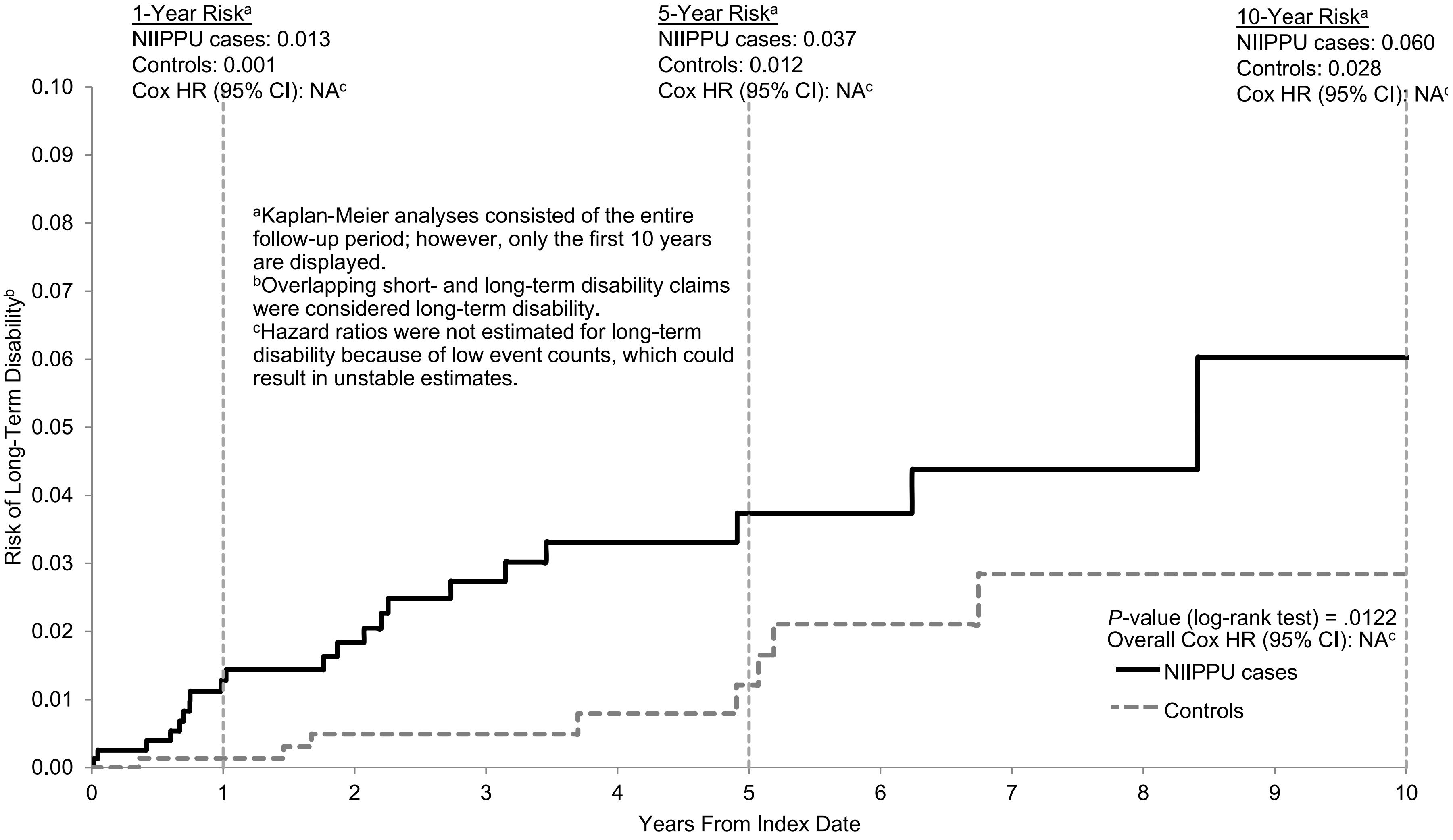

Patient counts:

NIIPPU cases:	776	631	471	366	281	219	170	114	74	49	35
Controls:	776	647	488	394	298	228	173	120	78	49	31

CI, confidence interval; HR, hazard ratio; NIIPPU, non-infectious intermediate, posterior, or panuveitis.

eFigure 5. Risk of Workforce Absence: Short-Term Disability

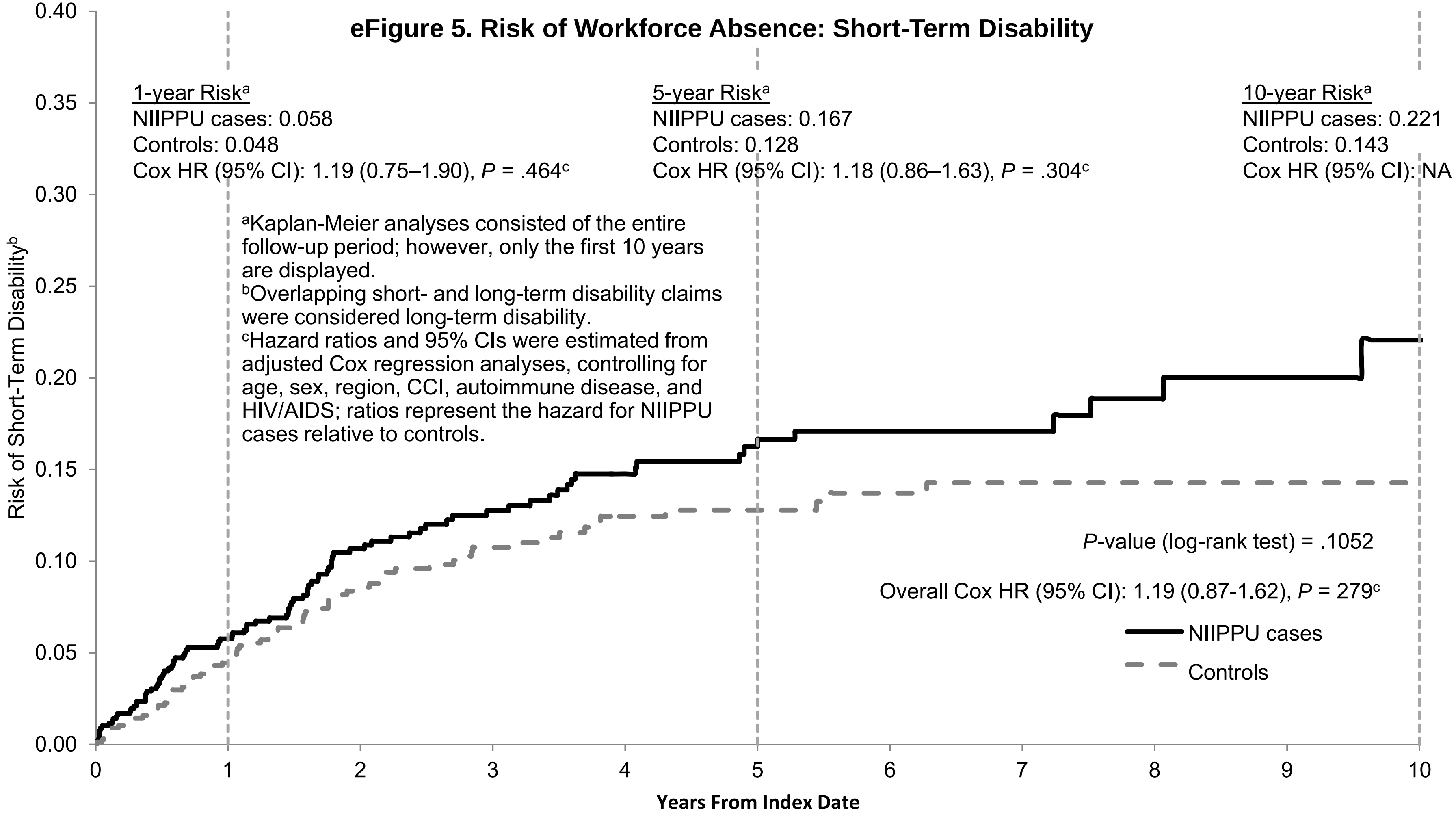

Patient counts:

NIIPPU:											
	776	603	429	334	258	201	159	109	72	49	34
Controls:											
	776	618	458	363	276	215	165	121	80	50	30

AIDS, acquired immunodeficiency syndrome; CI, confidence interval; HIV, human immunodeficiency virus; HR, hazard ratio; NIIPPU, non-infectious intermediate, posterior, or panuveitis.
