# Supplementary material for: Direct and indirect resource use, healthcare costs and work force absence in patients with non‐infectious intermediate, posterior or panuveitis
Source: Acta Ophthalmol. 2016 Mar 2;94(5):e331–9. doi: 10.1111/aos.12987 (PMC5069656; doi:10.1111/aos.12987)
Supplement: Supplementary file 2 — Table S1. Comparison of comorbidity profiles: full niippu samplesa. Table S2. Comparison of comorbidity profiles: prevalent sample, persistent casesa. [file AOS-94-e331-s002.pdf]

eTable 1. Comparison of Comorbidity Profiles: Full NIIPPU Samples[a]

	Utilization and Cost Analysis (Prevalent Population)		Work Loss Analysis (Incident Population)	
	**NIIPPU Cases (N = 705)**	**Controls (N = 705)**	**NIIPPU Cases (N = 776)**	**Controls (N = 776)**
Select ocular complications, %				
Retinal detachments	4.3%*	0%	2.6%*	0%
Retinal disorders	11.9%*	0.1%	9.3%*	0%
Glaucoma	7.4%*	1.6%	6.6%*	1.2%
Cataract	6.0%*	0.4%	4.9%*	0.8%
Visual disturbances	11.1%*	0.7%	10.4%*	0.5%
Blindness and low vision	2.1%*	0%	1.9%*	0%
Autoimmune comorbidities, n (%)				
Spondyloarthritis	1.0%*	0.1%	1.4%*	0.1%
Sarcoidosis	3.4%*	0.1%	1.7%*	0
Behcet's syndrome	0.3%	0%	0.1%	0
Multiple sclerosis	2.0%*	0.3%	1.2%*	0.1%
Systemic vasculitis	1.8%*	0.1%	1.9%*	0%
Inflammatory bowel disease	0.9%	0.1%	0.6%	0.4%
Vitiligo	0%	0.1%	0%	0%
Systemic lupus erythematosus	1.1%*	0%	0.8%	0.3%
Sjögren's syndrome	0.3%	0%	0.1%	0.1%
Relapsing polychondritis	0.4%	0.1%	0.3%	0%
Rheumatoid arthritis	2.4%*	0.4%	1.5%	0.6%
Psoriasis	1.1%	0.6%	1.0%	0.9%
CCI,[b] mean (SD)	0.9 (2.0)*	0.1 (0.6)	0.8 (1.8)*	0.2 (0.7)

CCI, Charlson Comorbidity Index; ICD-9, International Statistical Classification of Diseases, 9th edition; NIIPPU, non-infectious intermediate, posterior, or panuveitis; SD, standard deviation.
[a]Comorbidities were defined using ICD-9 codes.
[b]The CCI included 17 conditions identified using the ICD-9 diagnosis codes reported by Romano et al. (1993).
*Statistically significant difference (*P* <.05) between NIIPPU cases and controls without uveitis (McNemar's test for categorical variables and Wilcoxon signed rank tests for continuous variables).

Romano PS, Roos LL, & Jollis JG (1993): Adapting a clinical comorbidity index for use with ICD-9-CM administrative data: differing perspectives. J Clin Epidemiol **46**: 1075–1079.

eTable 2. Comparison of Comorbidity Profiles: Prevalent Sample, Persistent Cases[a]

	Utilization and Cost Analysis (Prevalent Population)	
	Persistent NIIPPU Cases (N = 112)	Controls (N = 112)
Select ocular complications, %		
Retinal detachments	5.4%*	0%
Retinal disorders	21.4%*	0%
Glaucoma	15.2%*	1%
Cataract	11.6%*	0%
Visual disturbances	12.5%*	2%
Blindness and low vision	0.9%	0%
Autoimmune comorbidities, %		
Spondyloarthritis	3.6%*	0%
Sarcoidosis	8.9%*	0%
Behcet's syndrome	0.9%	0%
Multiple sclerosis	3.6%*	0%
Systemic vasculitis	7.1%*	1%
Inflammatory bowel disease	2.7%	0%
Vitiligo	0%	0%
Systemic lupus erythematosus	3.6%*	0%
Sjögren's syndrome	1.8%	0%
Relapsing polychondritis	1.8%	0%
Rheumatoid arthritis	10.7%*	0%
Psoriasis	3.6%*	0%
CCI,[b] mean (SD)	0.75 (1.7)*	0.21 (0.74)

CCI, Charlson Comorbidity Index; ICD-9, International Statistical Classification of Diseases, 9th edition; NIIPPU, non-infectious intermediate, posterior, or panuveitis; SD, standard deviation.
[a]Comorbidities were defined using ICD-9 codes.
[b]The CCI included 17 conditions identified using the ICD-9 diagnosis codes reported by Romano et al. (1993).
*Statistically significant difference (*P* <.05) between NIIPPU cases and controls without uveitis (McNemar's test for categorical variables and Wilcoxon signed rank tests for continuous variables).

Romano PS, Roos LL, & Jollis JG (1993): Adapting a clinical comorbidity index for use with ICD-9-CM administrative data: differing perspectives. J Clin Epidemiol **46**: 1075–1079.
